# The Human Impact on All Soil-Forming Factors during the Anthropocene

**DOI:** 10.1021/acsenvironau.1c00010

**Published:** 2021-09-13

**Authors:** Ishai Dror, Bruno Yaron, Brian Berkowitz

**Affiliations:** Department of Earth and Planetary Sciences, Weizmann Institute of Science, Rehovot 76100, Israel

**Keywords:** Human impact, Soil properties, Environmental implications, Land use, Contamination, Soil quantity and quality, Agricultural sustainability, Climate change

## Abstract

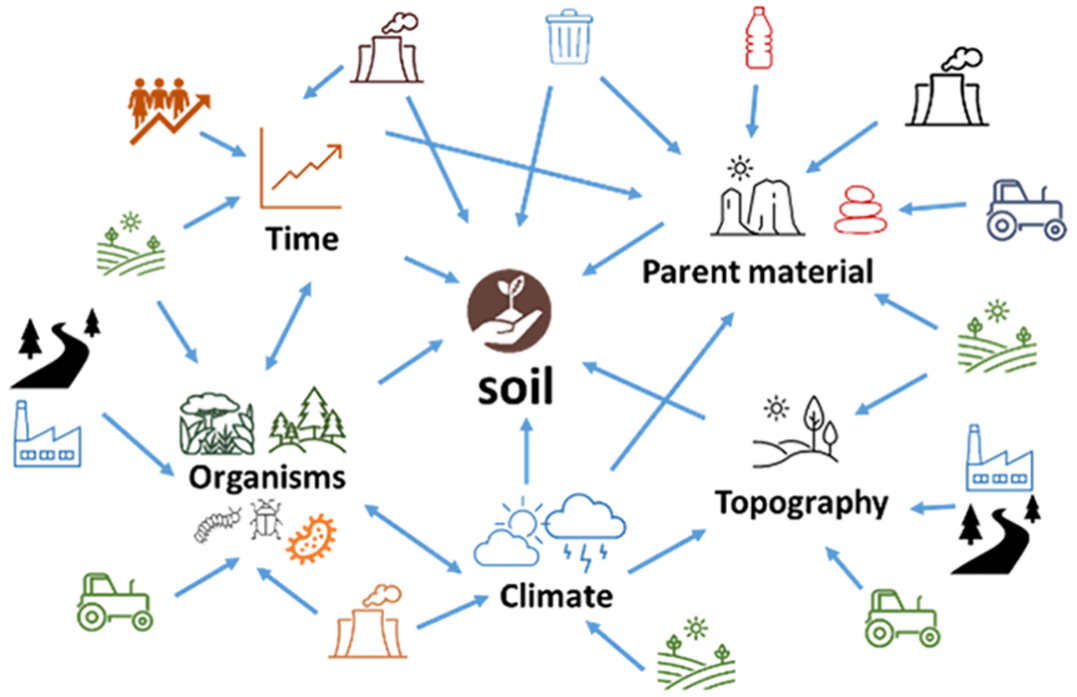

Soil—the thin outer skin of the Earth’s land—is a critical and fragile natural resource. Soil is the basis for almost all global agriculture and the medium in which most terrestrial biological activity occurs. Here, we reconsider the five forming factors of soil originally suggested more than a century ago (parent material, time, climate, topography, and organisms) and updated over the years to add human activity as the sixth forming factor. We demonstrate how present anthropogenic activity has become the leading component influencing each one of the original forming factors. We thus propose that, starting from the Anthropocene, human activity should no longer be considered as a separate forming factor but rather a main driving force of each of the five original ones. We suggest that the importance of soil and the strong direct and indirect effects of anthropogenic factors on soil-forming factors should be considered together to ensure sustainability of this critical resource.

## Introduction

Throughout the development of soil science, many different definitions of “soil” have been suggested. However, in all cases, it has always been clear that the sustainability and prosperity of life depend on the quality of soils.

The so-called “soil-forming factors” were first described by Dokuchaev^1^ and Darwin^2^ and developed further by other forefathers of soil science, including Hilgard^3^ and Jenny.^4,5^ The five soil-forming factors accepted by the soil science community are (i) parent material, (ii) time, (iii) climate, (iv) topography and relief, and (v) organisms. Soil formation is usually considered to be a long-term process. It is noted that these factors are not independent but rather show complex and high-order interactions that vary over time.

Almost a century after the first formal definition of the soil-forming factors, Yaalon and Yaron^6^ argued that human-induced changes in soil-forming processes should be considered as an integral, independent factor; this should be included as another (sixth) recognized forming factor, termed metapedogenesis, which is the result of anthropogenic activity on soil. Richter and Yaalon^7^ and recently Richter^8^ refer to this factor as anthropedogenesis, elegantly explaining the concept and reviewing the literature related to its development. Anthropogenic effects and their impact on the rate of soil modification were recognized subsequently as an independent factor.^7,9−13^

Here, we follow Yaalon and Yaron^6^ and their definition of metapedogenesis by proposing that, because anthropogenic effects have become so significant and so influential, they are often not only an additional factor or “disturbing agent” but also actually profoundly affect each of the five original soil-forming factors. We note that here we examine processes that are active currently. We do not claim that anthropogenic activities have changed the total soil inventory, nor that all global soils formed over eons are profoundly affected by human activities. To justify this argument, we briefly discuss each of the original soil-forming factors and include examples of how humans are domesticating and disrupting soils and how anthropogenic effects have become the most dominant influences on each of them.

## The Five Soil-Forming Factors in the Anthropocene

### Parent Material

**Figure 1:**
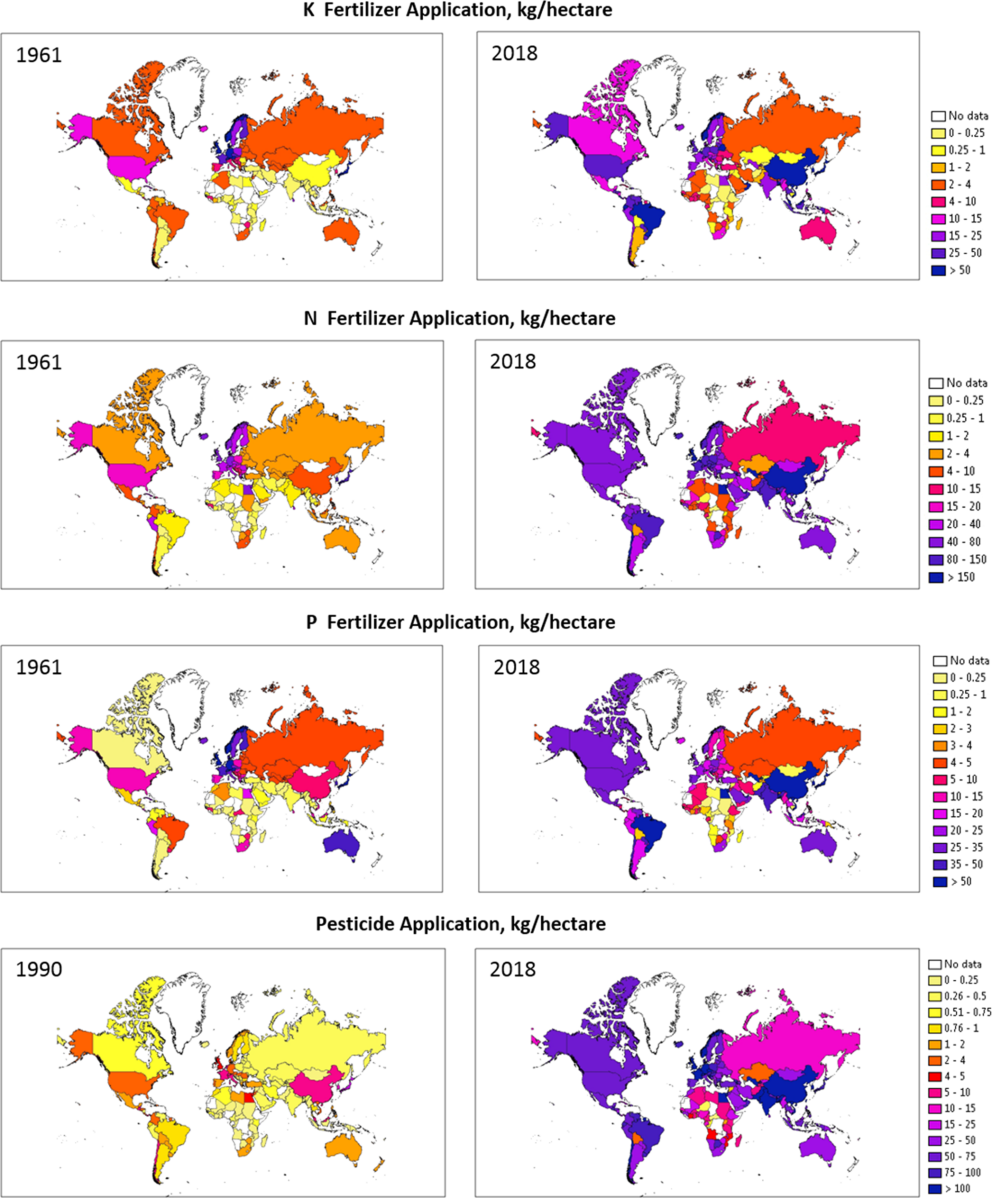
Global distribution of total potash fertilizers, total nitrogen fertilizers, total phosphate fertilizers, and total pesticides, per area of cropland (kg/ha), based on data freely available from the Food and Agricultural Organization of the United Nations.^18^

In the past, the soil composition was derived from its parent geological material. Parent material is a combination of bedrock weathered in place and geologic material transported by wind, water, ice, and gravity, supplying together the mineral components of the soil. However, more recently, massive anthropogenic activity has strongly influenced soil composition, adding new anthropogenic sources and changing the composition of many current natural sources. In fact, the (dry basis) mass of human-made materials that are comparable to soil parent material, including concrete, aggregates (mostly gravel and sand), and bricks, was reported^14^ recently to reach around 90% of the global biomass, thus emphasizing the staggering extent of anthropogenic materials being added to the pool of potential soil components. Moreover, many transport processes that are described hereafter (e.g., dams that change river transport, wind dust composition) are influenced strongly by human activities. New transport processes that diversify soil composition, like mixing different horizons by advanced machinery and moving large amounts of soil, have become common practice.^15−17^ Another major effect on soil composition is the introduction of soil amendments that in recent decades has also become common. Such amendments include composting, fertilizing, liming, and deposition of treated sewage sludge. Figure 1 demonstrates this trend by showing the increasing amount of fertilizers and pesticides applied to cropland around the world.

It is important to emphasize that addition of synthetic fertilizers and pesticides started only following their development less than 100 years ago. Figure 1 further shows that the amounts of amendments applied to cropland increased by more than an order of magnitude when levels from 1961–2018 were compared. The large impact of anthropogenic activity, in general, and soil amendments, in particular, on the soil composition should be considered, keeping in mind that (i) the total amount of cropland grew dramatically by more than 300% between 1820 and 2015, and (ii) the share of land used for agriculture is above 30% of the total land area in most parts of the world.^19^ It is further noted that many indirect and complex interactions, originating from processes that are related to the other forming factors, strongly influence parent material. For example, enhanced biological activity through agricultural practices can enhance weathering of parent material.

In addition, pollution processes such as acid rain and (anthropogenically generated) dust deposition (for example, from fly ash following burning of fossil fuels) can deplete or enrich substantially the soil mineral metrics derived originally from the parent material. A recent example of lead (Pb) contamination in urban soil^20^ in North Carolina, USA, demonstrated how Pb originating from human-made leaded gasoline and paints altered the upper soil layers. Lead concentrations high above geogenic background levels were detected in the upper 50 cm of the soil, demonstrating anthropogenic Pb enrichment. In fact, both the World Reference Base for Soil Resources^21^ and the USDA Soil Taxonomy^22^ acknowledge anthropogenic soils (often-termed anthrosols, anthroposols, antropozem, or anthropic soils^23^) and offer some classification for them, as detailed further in the Environmental Implications and Extent of Human Influence section below.

### Time

**Figure 2:**
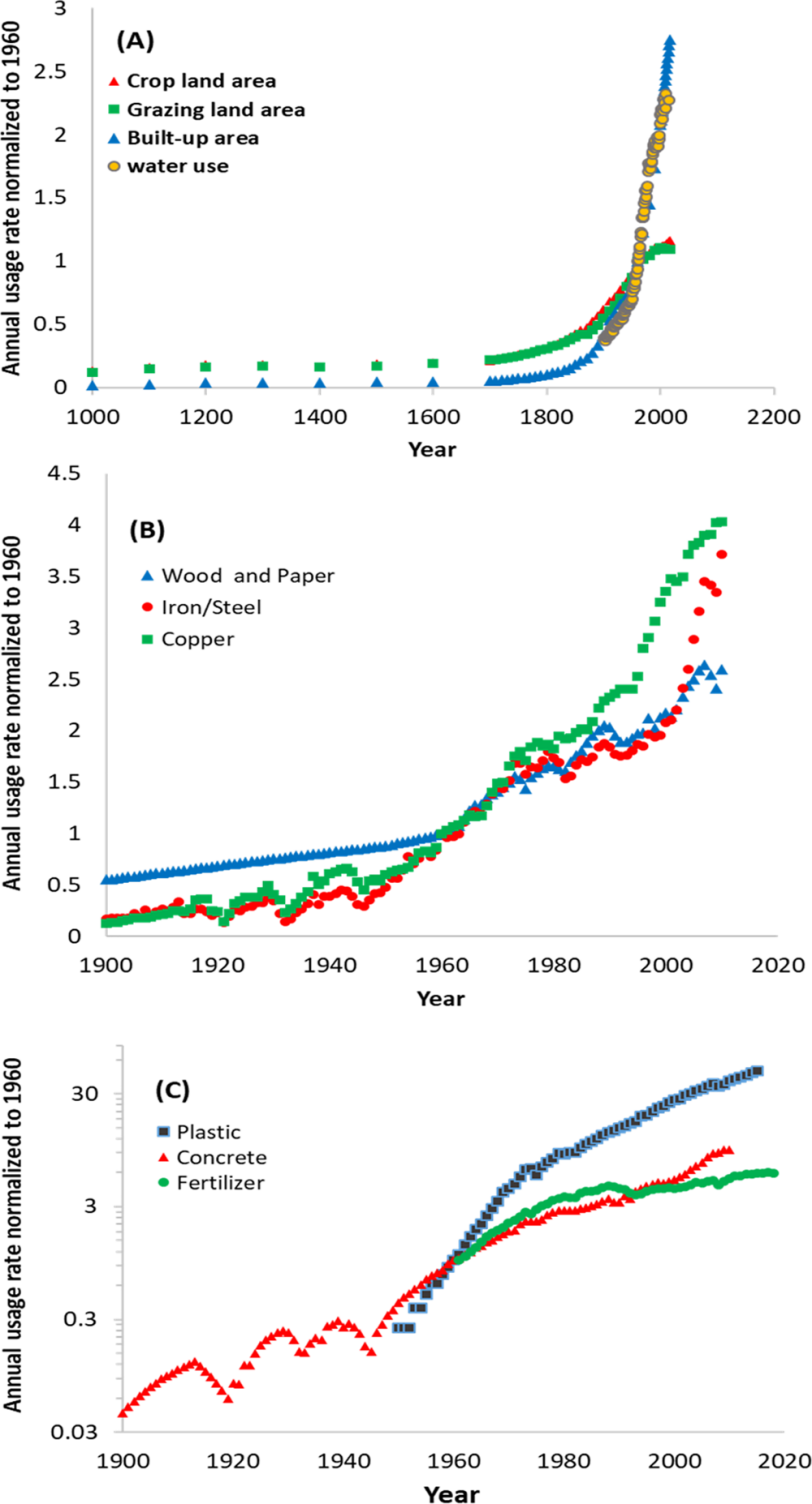
Changes in annual global usage/production of selected (A) natural resources, (B) natural products, and (C) anthropogenic products based on data available from the following sources: for water use^29^ cropland, grazing, and built-up area;^19^ wood and paper, iron/steel, copper, concrete,^30^ plastic,^31^ and fertilizer.^32^

Human activity is altering the time scale of soil formation. It has been argued that the anthropogenic factor is orders of magnitude faster than the “natural” processes that affect soil formation (hundreds of years vs hundreds of thousands and even millions of years, respectively^24^). It is noted that there are also several fast, natural processes, like landslides or flood events; although many of them are attributed to climate change, they also involve anthropogenic factors. In general, anthropogenic processes are on the order of hours (e.g., pollution accidents) to days (e.g., deep plowing, liming, and fertilizing) to years (e.g., terracing, landfill operation, or land use change). Figure 2 demonstrates the very fast recent changes (termed the “great acceleration”^25^) in land and water use (Figure 2A), consumption of natural resources (Figure 2B), and production of anthropogenic matter (Figure 2C) that is often released to the environment at the end of use. In Figure 2A, water and land use show a ∼5-fold increase within the last 100 years. Similarly, amplified exploitation of selected natural resources (wood, iron, and copper) by a factor of 6–10 is demonstrated for the same period (Figure 2B). The production of selected anthropogenic substances that are manufactured in high volumes is depicted by Figure 2C; for this class of substances, the production jumped by ∼2 orders of magnitude in the last century. Soil is the ultimate sink for most of these substances, which are added to the upper soil layers at high rates and change the composition of the matrix and its properties. In all cases, when considering the change compared to the soil formation rate, it is clear that human activity is impacting soil composition and structure at a much higher rate than the natural process.

Moreover, Pimentel et al.^26^ showed that anthropogenic soil erosion proceeds rapidly, significantly faster than soil formation rates; these authors indicated that “nearly one-third of the world’s arable land has been lost” over a 40 year period. In a later study, Pimentel^27^ estimated that soil loss from land area is 10–40 times faster compared to the rate of soil renewal. Similar rates of soil erosion were reported by Montgomery,^28^ who found that erosion in conventionally plowed agricultural fields is 1–2 orders of magnitude faster than soil formation rates.

### Climate

Anthropogenic factors that lead to climate change are already well recognized, and current discussions are centered on better understanding and quantification of the multifactorial processes of anthropogenic activity that lead to climate change. The numerous publications, reports, and databases that link climate change to human activity touch on every aspect of this “soil-forming factor”. It is well established that human activity influences factors like surface temperature^33^ and precipitation distribution and amount, frequency, and amplitude of extreme climate events (e.g., hurricanes, floods, droughts).

Dry land is projected to cover half of the global land surface by the end of the 21st century, as a result of climate change that leads to its fast expansion.^34^ Glacial melting that exposes land covered by ice, as well as sea level rise that will cause land loss, are also reported frequently.^35^

The global impact of anthropogenic climate change on soil can be demonstrated by considering (i) the rate of soil erosion and (ii) the loss of soil organic carbon (SOC). Recently, Borrelli et al.^36^ examined model simulations based on three greenhouse gas emission scenarios and showed a substantial increase in average global soil erosion of 30, 51, and 66% by 2070, compared to 2015 levels for different IPCC fossil fuel usage scenarios. This increase was quantified as a soil loss of 56.1–71.6 Pg year^–1^ (and large variabilities were reported for these numbers), with the losses being linked to the predicted climate change.

The result showed by Borrelli et al.^36^ suggests that reduction of the expected soil erosion compared to current trends could be achieved if sustainable approaches toward fossil fuel usage will be adopted. In fact, the authors suggest that reduced soil erosion could be an additional issue that should be considered by policy makers when considering the impact of climate change due to use of fossil fuels and the consequent emission of greenhouse gases.

The response of SOC to climate change is complex, and many factors are involved (e.g., temperature, humidity, biological activities, soil erosion) that have intrinsic feedback mechanisms. Chappell et al.^37^ estimated global terrestrial SOC erosion to be 0.3–1.0 Pg C year^–1^. A similar estimated SOC loss was suggested by Naipal et al.,^38^ who calculated global removal of 74 ± 18 Pg C for the period of 1850–2005 because of the combined effects of climate change, land use variation, and elevated levels of atmospheric CO_2_. It is noted that the fate of SOC is not clear, with some studies attributing nearly all eroded SOC to oxidization and addition to the atmosphere, and other studies suggesting that burial of eroded SOC can increase storage capacity and lead to carbon gain.^39^ Moreover, direct impacts of climate change (and other factors like land cover change) on pedodiversity in the USA were reported,^40^ linking climate change to the disappearance of some soil types and alteration of properties of other soil types.

It is further noted that elevated levels of CO_2_ (one of the major indicators of anthropogenic climate change) can lead to chemical alteration of soil minerals on surface rocks. These processes involve very complex cascades of chemical reactions. A simplified example of such a process is given by the case of CO_2_ reaction with soil water, which decreases the pH of the pore water because of carbonic acid formation that can, in turn, enhance mineral dissolution.^41^ For example, Harvey et al.^42^ reported that exposure of geological material to high levels of CO_2_ can lead to dissolution of calcite, feldspar, and 1:1 phyllosilicates; similarly, olivine dissolution as a result of exposure to carbonic acid was reported by Köhler et al.^43^ It is noted that in deeper soil layers, CO_2_ levels are much higher (by up to 2 orders of magnitude).

### Topography and Relief

Human activity changes topography on large scales. For example, dams on rivers, road construction, and urbanization processes often alter topography. Grill et al.^44^ reported that 48% of all river volume has already been moderately to severely impacted by either flow regulation, fragmentation, or both, with this number increasing to 93% upon completion of all dams under construction or in planning at the time of writing the article. Another example of large-scale modification is the loss of sediments that were once transported through the Nile river and deposited on the flood plain and Egyptian delta. Today, more than 98% of the sediment is deposited at Lake Nasser,^45^ which has resulted in the loss of silt that is rich in silica, aluminum, iron, and other trace elements and low in nitrogen in the Egyptian delta. This loss has led to modification of the soil composition in the delta and, in turn, to intensified application of synthetic fertilizers.^45^

Engineered drainage control of large basins to reduce flooding risks and designed planting and vegetation growth are also greatly influencing topography. For example, Jaramillo and Destouni^46^ reported that 46 and 50% of the land areas in 100 major basins around the world, which cover 35% of the Earth’s land area, have been moderately and strongly, respectively, affected by anthropogenic flow regulation and irrigation. Desertification processes, too, are impacted strongly by human activity.^46^

In fact, anthropogenic terrestrial sediment deposits, often termed “legacy” or anthropogenic sediments, have been discussed often in the literature and present a broader view of this human impact. James^47^ presents several definitions of legacy sediments; the most general definition includes multiple human activities that lead to various sedimentary structures and textures (primarily alluvium or colluvium), processes, and depositional sites. In this definition, James^47^ includes a wide spectrum of anthropogenic activities that includes agricultural practices, land use changes like deforestation, milling, mining, and logging, and damming or rerouting of rivers and water ways. James^47^ also offers multiple examples from human history (from Roman times in Europe to European settlement in Australia and North America) and suggests that these legacy sediments could cover entire floodplains by a thick “young soil” layer.

Terraces are another example of anthropogenic engineering of surface topography, with history dating back 5000 years. Terraces are ubiquitous in hill slopes and mountains and can be found in many parts of the world including Asia, Europe, Middle East, North and South America, and Africa.^48^ Terraces often substantially modify the hydrology and biochemical cycles in and around their location;^49^ they reduce natural soil erosion and runoff and allow biomass accumulation and nutrient enrichment. Because of their global spread and their impact on large areas, they are a good example of the strong anthropogenic impact on regional topography.

Another major impact on the land topography is related to infrastructure facilities such as transportation (roads, rail tracks, parking lots) and mines. For example, by 2050, it is predicted that the global combined road and rail network length will grow by 60% compared to 2010. This will lead to a combined addition of 250,000–350,000 km^2^ of built surface area.^50^ In fact, it was reported^51^ that only 7% of Earth’s terrestrial surface has roadless patches that are larger than 100 km^2^. Global mining areas were recently mapped by Maus et al.^52^ and found to total 57,277 km^2^. These facilities have the potential to cause major disruption to natural topography. In this context, an example of the potential impact is the Amazon basin, where 21% of the land area is under mining leases.^53^

### Organisms

Human activities such as modern agriculture, land-use changes, and pollution are changing dramatically—over large scales—the flora and fauna of soil. For example, Ramankutty et al.^54^ reported that, by the year 2000, cropland and pasture covered 12 and 22%, respectively, of the Earth’s ice-free land surface. This means that more than one-third of the earth’s surface is impacted directly by agricultural activities. Even more strongly, Ellis and Ramankutty^55^ reported that, by the early 2000s, anthropogenic processes altered >75% of the global, ice-free terrestrial biosphere. Ellis and Ramankutty^54^ also found that only ∼11% of net primary production could be attributed to pristine natural processes unaffected by human activity, while almost 90% of the integrated terrestrial net primary production and 80% of tree cover on a global basis were considered anthropogenic biomass. Similar observations were reported by Imhoff et al.,^56^ who attributed one-third of the net primary production to human consumption. Human activities not only altered the biodiversity and control productivity parameters but also substantially reduced biomass on a global basis. Barnosky et al.^57^ reported that the current rate of mass extinction (termed “the Anthropocene mass extinction”) is stronger than at any other period in the last ∼540 million years, showing a decline in the estimated number of species by more than 75%. Elhacham et al.^14^ recently reported that humanity roughly halved the mass of plants (on a dry mass basis), when current levels were compared to those of the preagricultural revolution. These authors also reported that, currently, the total anthropogenic material (mostly concrete, aggregates, asphalt, and bricks) exceeds the global sum of biomass, thus providing another indication of the huge impact of human activity.

At the microbiome level, Leff et al.^58^ demonstrated how soil microbial communities in 25 grassland sites across the globe shifted in composition in consistent ways, as a response to elevated nutrient (mostly N and P) inputs that are related to human activities. The authors indicate a decrease in relative abundance of methanogenic archaea, oligotrophic bacterial taxa, and mycorrhizal fungi with increase in soil nutrients.

Most arable soil is already subject to “organism control” both in terms of (i) plants/crops grown in these areas and (ii) controlled microbial communities (by pesticides and supply of nutrients). Other factors like irrigation, deep plowing, burning of natural vegetation, and growth of genetically engineered crops are also contributing to the human impact/control on the “organism soil-forming factor”. In addition, indirect impacts like climate change and pollution emissions can also have strong impacts on the diversity and prosperity of organisms. One example of such an effect is the noticeable anthropogenic enhanced “greening” caused by strong vegetation growth in the northern extratropical zones.^59^ These authors report an increase in greening of ∼80% from 1982 to 2011, enhanced mainly by elevated levels of greenhouse gases in the atmosphere. Other studies refer to the same phenomenon, attributing the greening to temperature change, nitrogen deposition, and land use change.^60^ Thus, while organisms indeed play an important role (the fifth soil-forming factor listed here), we emphasize that their activity is currently affected significantly by anthropogenic activities. In this context, greening, for example, then impacts soil formation and properties.

## Environmental Implications and Extent of Human Influence

The implications of anthropogenic activities on soil have been discussed extensively for many years in numerous publications. For example, Dazzi and Lo Papa^23^ defined seven major types of anthropogenic soils, all induced by human activities: (1) soils that were altered because of land use changes, for example, Solonchaks developed from Cambisols in arid environments because of irrigation; (2) diagnostic soil horizons related mainly to long-term applications of organic matter or to wetland cultivation; (3) new parent material, which is a mixture of gathered mineral and/or organic materials resulting from landfills and other forms of accumulated waste originating from human activities; (4) deep soil disturbance, including deep plowing, trenches, excavations, pipelines, and construction sites without any distinguishable horizons; (5) landform changes, referring to rearrangement of the land topography applied mostly to improve agricultural practices (the most obvious example being terracing); (6) topsoil changes, which refer to the combined impact of process like land use change (tillage, deforestation), change in soil pH (liming), regulation of soil water content (irrigation and drainage), addition of nutrients (fertilization), and/or contamination; and (7) soil construction/reconstruction: soils that are produced for the need/opportunity to “reconstitute” and/or generate new soil, mostly for areas degraded by natural disaster or termination of industrial or military activities; another option is tailoring of soil properties for specific crop growing. In all of these, parent material is of minor importance and human impact plays the major role.

Here, we provide five examples that demonstrate some of the leading consequences of human impacts on the different soil-forming factors in the context of environmental implications. It is essentially impossible to provide simple quantification of anthropogenic impacts on soil formation, mostly because of the complexity and the numerous direct and indirect processes that are involved. However, below, we offer several examples that demonstrate the global scale of anthropogenic factors and how the magnitude of human impacts are as large as, and sometimes even larger than, that of natural processes. It is noted that these consequences are often the outcome of a combination of anthropogenic effects.

One fast and dramatic process induced by human activity is soil erosion. The accelerated loss of topsoil is so rapid and so extensive that a recent report of the Food and Agricultural Organization of the United Nations^61^ suggests that soil erosion should be considered as mining of a nonrenewable natural resource. The report also indicates that the erosion rates of arable or intensively grazed lands are 2–3 orders of magnitude faster than natural erosion rates, and that soil on hilly slopes will ultimately be exhausted under standard agricultural practice. Agricultural practice as major human impact was demonstrated by Wuepper et al.,^62^ who found significant differences in soil erosion rates between countries with joint borders despite their natural erosion rates being practically indistinguishable. Thus, for example, Wuepper et al.^62^ found a striking gap between Haiti, with soil erosion rates of >75 t ha^–1^ year^–1^, and the Dominican Republic, where the soil erosion rates were <25 t ha^–1^ year^–1^, along the two sides of the border within a few kilometers (although the rates on both sides of the border are very high). Enhanced erosion has many other consequences, such as reducing rooting space and water holding capacity, which by a feedback mechanism reduces soil productivity. Another result of soil erosion is loss of nutrients due to water or wind erosion of finer, more chemically active, soil fine particles (i.e., clays) that deplete the soil nitrogen and phosphorus and enrich the soil with the less productive sand fraction.^61^

A second factor that is deeply influenced by anthropogenic activities is the diminution of the soil organic carbon (SOC) fraction. SOC is one of the largest reservoirs of global carbon. It affects soil physical and chemical properties like soil structure, water storage capacity, particle agglomeration, soil pH, and the presence and availability of nutrients. The primary process that induces SOC depletion is the conversion of natural soil to agroecosystems, drainage of wetlands, plowing, biomass burning or removal, and climate change. SOC depletion is intensified (in terms of rate and scale) in soils susceptible to enhanced erosion, salinization, nutrient loss, compaction, acidification, and contamination.^61^

Lal et al.^63^ estimated that global SOC losses reach 78 ± 12 Gt of carbon, which can be attributed to “short-sighted farming practices”. Wei et al.^64^ reported a mean rate of SOC decrease of 1.31 ± 0.10 mg ha^–1^ year^–1^ when a forest is converted to agricultural land, with a mean turnover rate constant of 0.016 ± 0.002 year^–1^ (corresponding to a mean turnover time of 62 years).

An example of global sediment retention as a consequence of anthropogenic river impoundments was demonstrated by Vorosmarty et al.,^65^ who showed that >50% of basin-scale sediment flux in regulated basins is potentially trapped in artificial impoundments. Moreover, human-built reservoirs intercept 25–30% of the global sediment flux, or 4–5 Gt, annually.

Soil contamination is another process by which anthropogenic activities significantly alter soil properties. Here, too, the impact can be related to several of the soil-forming factors, and the implications of contamination are extensive. The contaminants are often toxic to the soil microbial population (and upon exposure also to humans), leading to changes in the natural fauna and flora. Increased concentrations of pollutants can alter soil composition, water repellency, adsorption capacity, and soil pH and redox properties. Point sources of contamination could lead to intensively contaminated “hot spots”. For example, Paya Perez and Rodriguez Eugenio^66^ reported that a recent survey of the European Commission Joint Research Centre identified 650,000 sites with (current or past) contamination activities and estimated a total of 2.5 million contaminated sites exist in Europe. Out of the 650,000 identified sites, ∼10% have already been remediated. The same survey indicated that for ∼60% of the soil contamination sites, mineral oil and heavy metals were the primary contaminants. Diffuse contamination is a second mode of contaminant spreading, affecting large areas on a global scale. The leading contamination processes for this mode are atmospheric deposition, agriculture practices, and flood events. An example of the magnitude of the polluted area can be found in the work by Wei and Chen,^67^ who estimated that heavy metals have contaminated more than 20 million hectares, which account for a fifth of the total farmland in China. Dispersion of microplastics in soils in another source of widespread contamination. Recently, de Souza Machado et al.^68^ found that terrestrial microplastic pollution is 4–23 times greater than marine pollution. These authors further note that of almost 400 M tons yr^–1^ of plastic produced globally, about one-third is ultimately deposited in soils or freshwater.

The last example relates to changes in soil biodiversity. Soil biota supply a wide range of essential services, such as regulating nutrient cycles, modifying soil physical structure and soil water regimes, controlling the dynamics of soil organic matter and carbon sequestration, and enhancing the amount and efficiency of nutrient acquisition. Wagg et al.^69^ reported that a decline in soil biodiversity disturbs numerous ecosystem functions, for example, above-ground plant diversity, cycling and retention of nutrients, and organic matter decomposition. Increased agricultural practices (e.g., selective growth of crops/plants, fertilizer application, regulating pH, tillage, use of pesticides, herbicides, and pollution) were reported to reduce soil biodiversity.^61,70^ Similarly, the conversion of natural habitats of soil biota to agricultural land was linked by Bardgett and van der Putten^71^ to changes in soil physical properties like soil temperature, pH, and water-holding characteristics.

## Conclusions

We argue that direct and indirect anthropogenic activity has become the most influential factor currently affecting each of the five original soil-forming factors, to the point that human impacts are not an additional factor, but actually a major (and often dominant) control on all five of these original soil-forming factors.

**Scheme 1:**
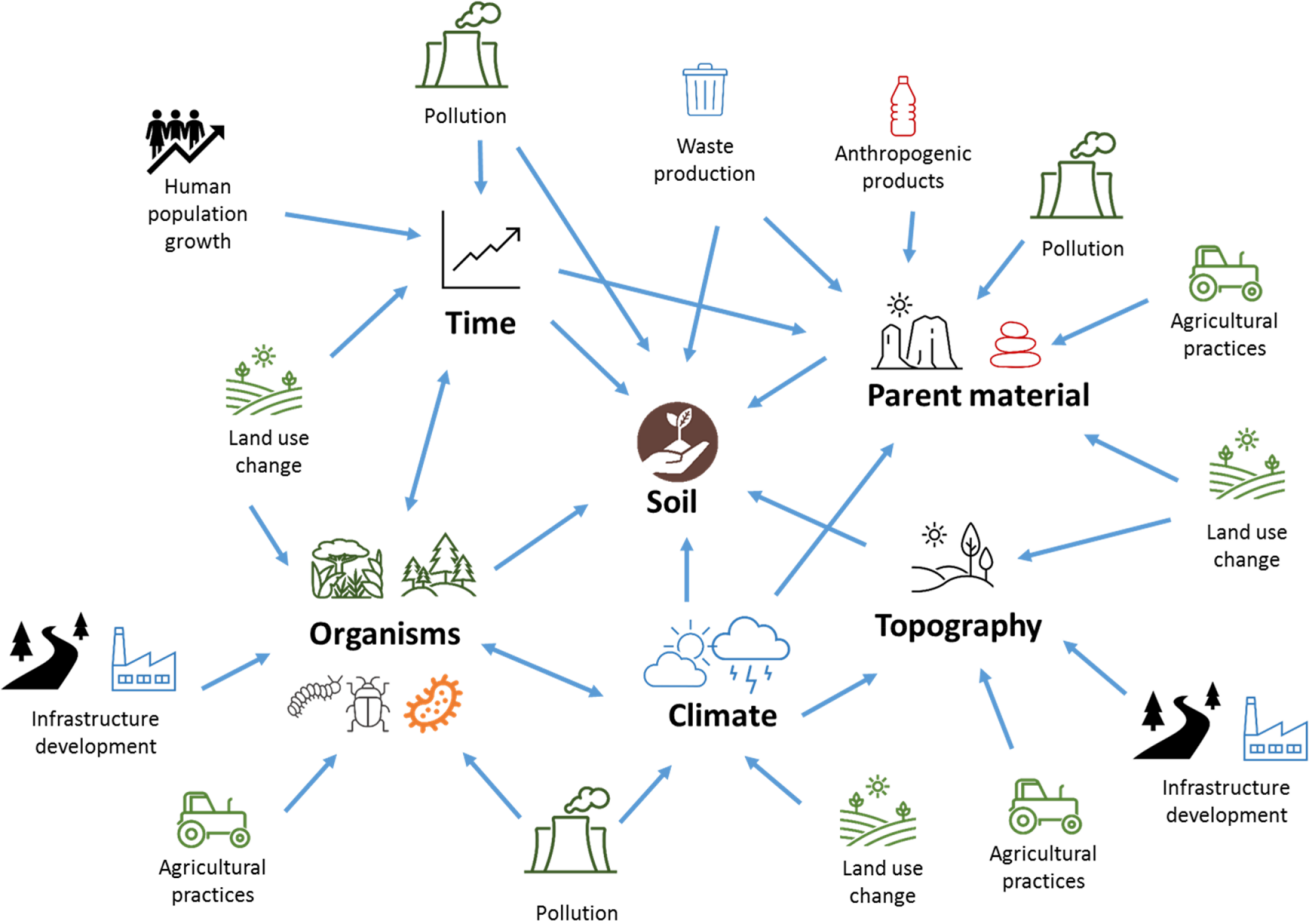
Schematic Illustration of Anthropogenic Impacts on the Five Soil-Forming Factors

Scheme 1 presents a simplified schematic representation based on some of the anthropogenic processes discussed above, showing how they are involved in the fast and drastic changes that alter each of the five soil-forming factors. Scheme 1 also highlights the different feedback mechanisms among the anthropogenic processes and the soil-forming factors. For example, climate change has an impact on all other soil-forming factors, and is influenced in a feedback by many anthropogenic processes (e.g., emission of pollution, industrial and agricultural activities and land use changes).

This analysis offers an additional perspective on the geological epoch known as the Anthropocene. Because of the fundamental importance of soil for human and ecosystem sustainability, and because soil is the matrix upon which most of the world’s food is grown and cities and roads are developed, human activities and their consequent impact on soil should be monitored and managed more carefully.
